# A Cure for Styes

**Published:** 1885-03

**Authors:** 


### A CURE FOR STYES.

Among the most troublesome and often noticed eye affections are what are known as hordelum, or common stye. Dr. Louis Fitzpatrick, in the *Lancet*, differs from some of his professiónal brethren, who persist in ordering the application of poultices, bathing with tepid water, &c. These no doubt do good in the end, but such applications have the great disadvantage of prolonging the career of these unsightly sores, and encourage the production of fresh ones. Dr. Fitzpatrick has found, after many trials, the local application of tincture of iodine exert a well-marked influence in checking the growth. This is by far preferable to the nitrate of silver, which makes an unsightly mark, and often fails in its object. The early use of the iodine acts as a prompt abortive. To apply it, the lids should be held apart by the thumb and index finger of the left hand, while the iodine is painted over the inflamed papilla with a fine camel hair pencil. The lids should not be allowed to come in contact until the part touched is dry. A few such applications in the twenty-four hours is sufficient.

